# Genotype characterization of Epstein–Barr virus among adults living with human immunodeficiency virus in Ethiopia

**DOI:** 10.3389/fmicb.2023.1270824

**Published:** 2023-10-31

**Authors:** Kidist Zealiyas, Seifegebriel Teshome, Aklilu Feleke Haile, Christoph Weigel, Ayinalem Alemu, Wondwossen Amogne, Getnet Yimer, Tamrat Abebe, Nega Berhe, Elshafa Hassan Ahmed, Robert A. Baiocchi

**Affiliations:** ^1^Aklilu Lemma Institute of Pathobiology, Addis Ababa University, Addis Ababa, Ethiopia; ^2^Ethiopian Public Health Institute, Addis Ababa, Ethiopia; ^3^Department of Microbiology, Immunology and Parasitology, Addis Ababa University, Addis Ababa, Ethiopia; ^4^Comprehensive Cancer Center, The James Cancer Hospital and Solove Research Institute, The Ohio State University, Columbus, OH, United States; ^5^Division of Hematology, Department of Internal Medicine, College of Medicine, The Ohio State University, Columbus, OH, United States; ^6^Department of Internal Medicine, College of Health Sciences, Addis Ababa University, Addis Ababa, Ethiopia; ^7^Centre for Innovative Drug Development and Therapeutic Trials for Africa (CDT-Africa), College of Health Sciences, Addis Ababa University, Addis Ababa, Ethiopia; ^8^Department of Genetics, Penn Center for Global Genomics & Health Equity, Perelman School of Medicine, University of Pennsylvania, Philadelphia, PA, United States

**Keywords:** Epstein–Barr virus, EBNA1, EBNA2, EBNA3C, Ethiopia, HIV status

## Abstract

**Background:**

Epstein–Barr virus (EBV) is a human lymphotropic herpesvirus with a causative agent in cancer. There are two genotypes of EBV (EBV genotype 1 and EBV genotype 2) that have been shown to infect humans. This study aimed to characterize the EBV genotype among people with human immunodeficiency virus (PWH) and HIV-negative individuals in Ethiopia.

**Methods:**

DNA was extracted from peripheral blood mononuclear cells (PBMCs). Conventional polymerase chain reaction (cPCR) targeting *EBNA3C* genes was performed for genotyping. A quantitative real-time PCR (q-PCR) assay for EBV DNA (*EBNA1* ORF) detection and viral load quantification was performed. Statistical significance was determined at a value of *p* < 0.05.

**Result:**

In this study, 155 EBV-seropositive individuals were enrolled, including 128 PWH and 27 HIV-negative individuals. Among PWH, EBV genotype 1 was the most prevalent (105/128, 82.0%) genotype, followed by EBV genotype 2 (17/128, 13.3%), and mixed infection (6/128, 4.7%). In PWH, the median log10 of EBV viral load was 4.23 copies/ml [interquartile range (IQR): 3.76–4.46], whereas it was 3.84 copies/ml (IQR: 3.74–4.02) in the HIV-negative group. The EBV viral load in PWH was significantly higher than that in HIV-negative individuals (value of *p* = 0.004). In PWH, the median log10 of EBV viral load was 4.25 copies/ml (IQR: 3.83–4.47) in EBV genotype 1 and higher than EBV genotype 2 and mixed infection (*p* = 0.032).

**Conclusion:**

In Ethiopia, EBV genotype 1 was found to be the most predominant genotype, followed by EBV genotype 2. Understanding the genotype characterization of EBV in PWH is essential for developing new and innovative strategies for preventing and treating EBV-related complications in this population.

## Introduction

Epstein–Barr virus (EBV), known as Human herpesvirus 4, is one of the most common human oncogenic viruses, with more than 90% of adults infected world-wide (Kimura et al., 2013; Mentzer et al., 2022). In developing countries, primary infection by EBV most often occurs within the first few years of life and is usually asymptomatic. When this primary infection occurs in adolescents or adults, it can cause the self-limiting infectious mononucleosis syndrome (Williams and Crawford, 2006).

Transmission of the virus occurs predominantly through exposure to infected saliva and persists as a latent infection in the human B-cell compartment. However, EBV can also spread through blood and semen during sexual contact, blood transfusions, and organ transplantations (Valachis and Kofteridis, 2012). The infected memory B lymphocytes can migrate back to the tonsils, where they can induce more viral replication, spreading and infecting other B lymphocytes (Thorley-Lawson and Gross, 2004; Young and Rickinson, 2004). Lifelong persistence occurs through the establishment of latent reservoirs in B cells and periodic reactivation that primarily occurs in oropharyngeal tissues (Thompson and Kurzrock, 2004).

EBV usually causes limited serious consequences in immune-competent individuals; however, in immune deficient patients, the virus is associated with a wide spectrum of malignancies such as nasopharyngeal carcinoma (NPC), gastric carcinoma (GC), Hodgkin’s lymphoma (HL), non-Hodgkin’s lymphoma (NHL), extra-nodal natural killer (NK) cell/T-cell lymphomas, and immunodeficiency-associated lymphoproliferative disorders (Miller, 1996; Maeda et al., 2009). For HIV-positive individuals in the era of highly active antiretroviral therapy, the incidence of AIDS-defining malignancies such as NHL has fallen while the incidence of non-AIDS-defining malignancies such as Hodgkin lymphomas is rising.

The EBV genome is 172 kilobase pairs in size and encodes for more than 85 genes (or open reading frames, ORF) that express proteins according to the phase of the viral life cycle (Smatti et al., 2018). Six EBV nuclear antigens (*EBNA1, EBNA2, EBNA3A, EBNA3B, EBNA3C*, and *EBNA*-leader protein) and three latent membrane proteins (*LMP1*, 2A, and 2B) are among the ORFs that regulate viral life cycle and oncogenic activity (Houldcroft and Kellam, 2015). *EBNA3A, EBNA3B, and EBNA3C are ORFs* arranged in tandem in the EBV genome and encode for transcription factors (Bazot et al., 2014). *EBNA3A* and *EBNA3C* are essential for EBV’s capacity to transform human B cells into lymphoblastoid cell lines (Maruo et al., 2011). *EBNA3C* can act as a transcription factor to regulate viral and host gene expression as well as a mediator driving protein–protein interaction with protein targets such as the RB tumor suppressor (Allday et al., 2015; Ohashi et al., 2021).

There are two main EBV genotypes that have been detected in humans: EBV genotype 1 and EBV genotype 2. These two genotypes were distinguished based on differences in the EBV nuclear antigen sequences (*EBNA2*, -3A, -3B, and -3C; Adldinger et al., 1985; Sample et al., 1990; Tzellos and Farrell, 2012). There are a variety of methods for genotyping EBV and differentiating its variants. *EBNA3A*, 3B, 3C, and *EBNA2* have all been used for EBV genotyping, but the most studied and approved method is to use *EBNA3C* (Snudden et al., 1995; Edwards et al., 1999). *EBNA1* and different regions of the *LMP1* gene sequences can also be used to classify viral variants (Correia et al., 2017). However, *EBNA3C* is still the most common method for genotyping EBV, and it can distinguish between different pathogenic and geographical patterns of the virus (Lin et al., 1993).

The geographical distribution of EBV types reveals that EBV genotype 1 is the most frequently seen worldwide, with its highest prevalence in Europe, Asia, North and South America, and other parts of the world. Papua New Guinea, Central Africa, and Alaska are where EBV genotype 2 is more common (Giron et al., 2013; Zanella et al., 2019). The biological characteristics of the two genotypes also differ; EBV genotype 1 is more effective at immortalizing B cells, while EBV genotype 2 has a higher lytic ability (Griffiths et al., 2000; Klemenc et al., 2006).

The EBV genotype among people with HIV (PWH) showed that EBV genotype 1 was predominant, followed by EBV genotype 2 and mixed infection (Pereira et al., 2022; Wan et al., 2023). Some studies have found that EBV genotype 2 or mixed infection is more frequently found in immunosuppressed patients, indicating that host immune function could influence the reactivation or latency of EBV infection (Chang et al., 2009; Neves et al., 2017). However, it is yet unknown how many EBV variants can exist in a single person or whether an individual with a history of infection is immune to multiple variants (Farrell, 2015).

Exploring the genotype diversity of EBV is important to achieve a better understanding of EBV biology as well as the relationship between EBV genotype variation and EBV-associated diseases (Cirac et al., 2018). There is limited data on the genotype characterization of EBV among PWH in Ethiopia. Hence, this study aimed to determine EBV genotyping among PWH and HIV-negative individuals in Ethiopia.

## Materials and methods

### Study design

A cross-sectional study was conducted at Tikur Anbesa Specialized Hospital (TASH) over a period of 1 year (March 2021 to 2022). The hospital is a tertiary care teaching hospital at Addis Ababa University, located in Addis Ababa, the capital city of Ethiopia. TASH has a diverse population of patients, including a large number of PWH on antiretroviral therapy (ART), and cases are referred to this tertiary care center from across the country.

### Isolation of peripheral blood mononuclear cells

Blood samples were collected in Acid Citrate Dextrose (ACD) tubes, and in accordance with the manufacturer’s instructions, lymphocytes were isolated using Ficoll-Paque PLUS (Global Life Sciences Solutions United States LLC) as described previously (Riedhammer et al., 2016). Briefly, blood samples were diluted 1:1 in 1X phosphate buffered saline (PBS) balanced salt solution (Life Technologies, United States), and then the diluted blood was overlayed on Ficoll-Paque PLUS solution. PBMCs were collected from the PBS-ficoll interface layer following centrifugation at 2000 rpm for 30 min at 20°C. To remove platelets, Ficoll-Paque PLUS, and any leftover plasma, PBMCs underwent washing procedures in a balanced salt solution. After counting viable PBMCs, they were placed in cryovials with 10% DMSO freezing medium and subjected to control rate freezing at −80°C. Cells were then shipped to Ohio State University in the United States, where DNA extraction was performed.

### DNA extraction

According to the manufacturer’s instructions, DNA was isolated from 5×10^6^ PBMCs in 200 μL of using the PBMCs (genomic) DNA was extracted using the QIAamp DNA Mini Kit (QIAGEN, Germantown, MD, United States; Qiagen, 2016). To minimize potential sources of contamination, we have worked in a clean environment and used sterile reagents and equipment. All extracted DNA samples were measured for purity and concentration using a Qubit 3.0 fluorometer (Life Technologies, Waltham, MA, United States) to ensure the quality of the sample, and they were then stored for further use at-80°C.

### EBV DNA detection and viral load quantification by *EBNA1*

The ViiA 7 real time qPCR machine (Applied Biosystems) was used to detect EBV using the *EBNA1* gene with an initial concentration of 10 ng of DNA. We tested 2, 10, and 20 ng gDNA concentrations in this assay. To ensure the assay will pick up enough copies of target DNA (EBV *EBNA1*) and avoid having a very small Ct value for the housekeeping gene (ACTB), we subsequently used a 10 ng of DNA per reaction. We also ran a denaturation curve after each qPCR assay to confirm reaction specificity and to verify that only the desired target was amplified. Signals were normalized to host genome DNA using primers specific for human *ACTB* (forward: CAGGCAGCTCGTA GCTCTTC, reverse: TCGTGCGTGACATTAAGGAG), and EBV DNA was quantified using primers specific to the EBV *EBNA1* locus (forward: TCATCATCCGGGTCTCC, reverse: CCTACAGGGTG GAAAAATGGC). The reaction was performed using 5 μL of 2× Fast SYBR Green Master Mix (Applied Biosystems), 0.25 μL of forward (10 μM) and 0.25 μL of reverse (10 μM) primers, 2.5 μL of distilled water, and 2 μL of 5 ng/μL DNA concentration with a total reaction volume of 10 μL. Real-time qPCR was performed with 40 cycles consisting of 95°C for 1 s, 60°C for 20 s, and 70°C for 30 s. The Raji cell line was used as a positive control, whereas the K-562 cell line was used as a negative control. These cell lines were obtained from the American Type Culture Collection (ATCC) and have been stored in the laboratory for a long time (ATCC numbers CCL-86 and CCL-243, respectively). Twelve standards of known DNA copy number for the *EBNA1* gene and *ACTB* locus at different concentrations were used to calculate the number of EBV copies per ml. The *EBNA1* gene standard was obtained from the *EBNA1* amplicon with accession number *EBNA1*-NC_007605.1. The ACTB gene is a housekeeping gene for normalizing the host genome DNA, and we have used actin beta obtained from *Homo sapiens* with the accession number NM_001101.5. On 384-well PCR plates, each sample was run in triplicate with the corresponding standard. To determine the EBV copy number, the CT value of the *EBNA1* gene was calculated and converted into EBV copies/ml. Genome copies per cellular genome elevated more than 2-fold above the negative control were considered EBV DNA positive.

### EBV genotyping by Epstein Barr nuclear antigen 3C

EBV genotype 1 and EBV genotype 2 were determined using standard PCR assays across type-specific regions of the *EBNA3C* gene as described previously (Sample et al., 1990). Conventional PCR is performed for the detection of EBV genotypes by the *EBNA3C* gene. A set of *EBNA3C* primers was used, including forward 5′-AGA AAG GGA GCG TGT GTT G-3′ and reverse 5’-GGC TCG TTT TTG ACG TCG G-3′.

This *EBNA3C* primer is designed for targeting the region of divergence between EBV genotype 1 (B95 coordinate 87,651–87,669) and EBV genotype 2 (B95-8 coordinate 87,803–87,783; (Kafita et al., 2018)). These coordinates are nucleotide sequences in the reference genome that have an accession number of NC_007605.1. PCR was performed in 20 μL using 1 μL of forward (10 μM) and 1 μL of reverse primers (10 μM), 10 μL 2X Platinum™ Direct PCR Universal Master Mix (Thermo Scientific, Baltic, UAB), 3 μL of distilled water, and 5 μL of DNA sample with a total concentration of 50 ng. We used B-95.8 *(ATCC VR-1492)* and Jijoye (*ATCC CCL-87*) cell line extracts as a reference for EBV genotypes 1 and 2, respectively. In all experiments, a negative control (without genomic DNA) was used. Thermal cycling was initiated at 98°C for 2 min, followed by 40 cycles including denaturation at 94°C for 15 s, annealing at 60°C for 15 s, and extension at 68°C for 20 s. Then, PCR-amplified products were separated on a 2% ethidium bromide-stained agarose gel and visualized using a Bio Rad ChemiDoc^™^ imaging system. Amplification products of 153 bp for EBV genotype 1 and 246 bp for EBV genotype 2 were used to distinguish between the two EBV genotypes.

### Statistical analysis

Statistical analysis was performed using IBM SPSS statistical software version 26 (SPSS Inc., Chicago, IL, United States). All variables were summarized using frequencies and proportions. Categorical variables were compared using the Chi-squared test. The Kolmogorov (0.158, *p* < 0.0001) and Smirnov tests (0.770, *p* < 0.0001) were employed to assess the normality of the EBV viral load. The result showed that the data was not normally distributed. Therefore, we used the median EBV viral load to compare HIV-positive and HIV-negative individuals. Besides, a nonparametric Kruskal-Wallis H test was used to compare medians among more than two groups, and a nonparametric two-tailed Wilcoxon rank-sum (Mann–Whitney U) test was used to compare two unpaired data sets. A value of *p* less than 0.05 was considered statistically significant.

## Results

### Socio demographic and clinical characteristics

In this study, a total of 155 EBV-positive study participants were included. Among them, 128 had been co-infected with both EBV and HIV, and the remaining 27 participants were HIV negative. Females were predominant in number (*n* = 81, 63.3%). The majority (*n* = 43, 33.6%) were over 48 years old, and most of them had completed secondary school (*n* = 52, 40.6%). Among HIV-negative individuals, more than half were females (*n* = 16, 59.3%%), nearly one-half (*n* = 12, 44.4%) were in the age group 18–27 years, and about half had completed a college diploma and above (*n* = 14, 51.9%; (Table 1)). Statistically, age and educational background are significantly associated with HIV status (*p* < 0.001).

**Table 1:** Demographic and clinical characteristics of study participants with HIV status.

Characteristics		HIV positive (*N* = 128)	HIV negative (*N* = 27)	*p* Value
Sex	Female	81(63.3%)	16(59.3%)	0.695
	Male	47(36.7%)	11(40.7%)	
Age	18–27	13(10.2%)	12(44.4%)	<0.001
	28–37	30(23.4%)	9(33.3%)	
	38–47	42(32.8%)	4(14.8%)	
	≥ 48	43(33.6%)	2(7.4%)	
Educational status	Illiterate	13(10.2%)	0(0.0%)	<0.001
	Primary school	45(35.2%)	3(11.1%)	
	Secondary school	52(40.6%)	10(37.0%)	
	College diploma and above	18(14.1%)	14(51.9%)	
HIV viral load	<1,000 RNA Copies/ml	115(89.8%)	-	
	>1,000 RNA Copies/ml	13(10.2%)	-	
CD4 Count	<200 cells/μl	21(16.4%)	-	
	≥ 200 cells/μl	101(78.9%)	-	
ART	1st line	89(69.5%)	-	
	2nd line	32(25.0%)	-	
	3rd line	5(3.9%)	-	
Regimen Type	ATV/r (PI based)	32(25.0%)	-	
	Darunavir (PI based)	5(3.9%)	-	
	DTG (INSTI based)	83(64.8%)	-	
	EFV/NVP (NNRTI based)	6(4.7%)	-	

ART, Anti-Retroviral Therapy; CD, Cluster of differentiation; HIV, Human Immunodeficiency Virus; “-”: Not applicable.

We assessed the association of demographic and clinical characteristics with EBV genotype. Our results showed that EBV genotype 1 was higher in age groups 18 to 27 years (*n* = 24, 96.0%). There was no difference between females and males in the type of EBV genotype infection. Among HIV-positive individuals (*n* = 128), EBV genotype 1, genotype 2, and mixed infections were detected in 105 (82.0%), 17 (13.3%), and 6 (4.6%), respectively. Moreover, among HIV-positive individuals with an HIV viral load greater than 1,000 RNA copies/ml (*n* = 13, 100%) and with CD4 count cells greater than 200 (*n* = 82, 81.2%), EBV genotype 1 was higher (Table 2).

**Table 2:** Demographic and clinical characteristics disaggregated by EBV genotype.

Characteristics		EBV 1	EBV 2	Mixed infection	Total	*p* Value
Age	18–27	24(96%)	1(4.0%)	0	25	0.643
	28–37	30(76.9%)	7(17.9%)	2(5.1%)	39	
	38–47	37(80.4%)	7(15.2%)	2(4.3%)	46	
	≥48	37(82.2%)	6(13.3%)	2(4.4%)	45	
Sex	Female	81(83.5%)	11(11.3%)	5(5.2%)	97	0.356
	Male	47(81.0%)	10(17.2%)	1(1.7%)	58	
HIV status	Negative	23(85.2%)	4(14.8%)	0	27	0.514
	Positive	105(82.0%)	17(13.3%)	6(4.7%)	128	
Most HIV Viral Load	NA	23(85.2%)	4(14.8%)	0	27	0.331
	<1,000	92(80.0%)	17(14.4%)	6(5.2%)	115	
	>1,000	13(100%)	0	0	13	
CD4 Count	<200	17(81.0%)	3(14.3%)	1(4.8%)	21	0.839
	≥200	82(81.2%)	14(13.9%)	5(5.0%)	101	
	Missed data	6(100%)	0	0	6	
	NA	23(85.2%)	4(14.8%)	0	27	

CD, Cluster of differentiation; HIV, Human Immunodeficiency; NA, Not Applicable.

### EBV genotypes in HIV-positive and HIV-negative individuals among study participants

**Figure 1:**
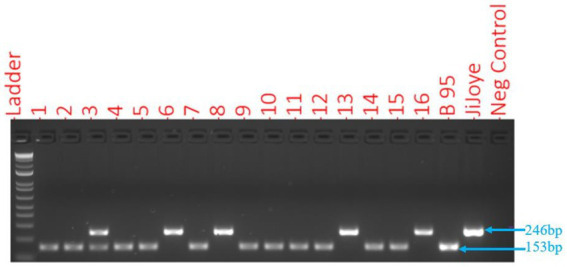
EBV genotyping by *EBNA3C-specific* primers with 153 bp for EBV genotype 1 and 246 bp for EBV genotype 2. Numbers 6, 8, 13, and 16 were EBV genotype 2, and number 3 had a mixed infection, while others were EBV genotype 1.

Of the 155 EBV-positive samples, most were EBV genotype 1 (82.6%), followed by EBV genotype 2 (13.5%) and mixed infections (3.9%). Genotyping of EBV based on different amplicon sizes by *EBNA3C*-specific primers (amplicon sizes 153 = type 1 and 246 bp = type 2) allowed us to distinguish EBV genotype 1 and genotype 2. Samples with mixed EBV infections were characterized by the presence of two amplicons (Figure 1).

### EBV genotype with HIV status

**Figure 2:**
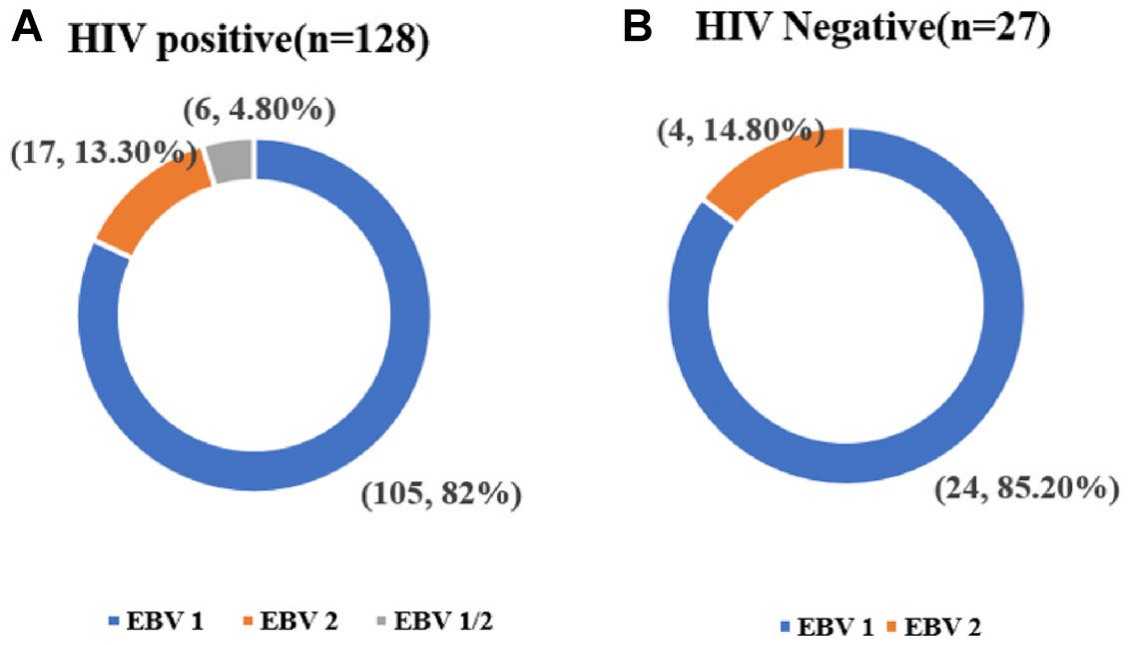
EBV genotype in HIV-positive and HIV-negative individuals. **(A)** In HIV-positive individuals. **(B)** In HIV-negative individuals.

Among the HIV/EBV coinfected individuals, EBV genotype 1 was the most prevalent genotype (*n* = 105/128, 82.0%), followed by the EBV genotype 2 genotype (*n* = 17/128, 13.3%), and mixed infection with both genotypes (*n* = 6/128, 4.7%; (Figure 2A)). In HIV-negative individuals with EBV infection, EBV genotype 1 accounted for 85.2% (*n* = 23/27) and EBV genotype 2 accounted for 14.8% (*n* = 4/27). Mixed infection with EBV genotype 1 and EBV genotype 2 was not found in HIV-negative participants (Figure 2B).

### EBV DNA detection and viral load in HIV-positive and HIV-negative individuals

**Figure 3:**
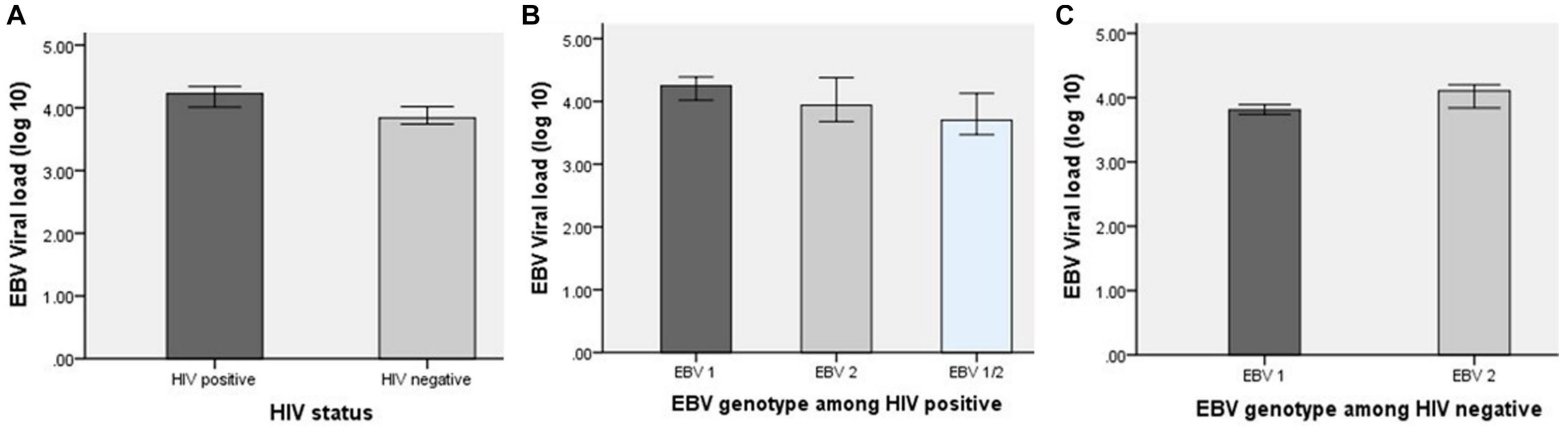
EBV viral load among HIV-positive and HIV-negative groups. **(A)** EBV viral load levels with HIV status (value of *p* = 0.004). **(B)** EBV viral load levels in HIV-positive individuals classified according to EBV genotype (value of *p* = 0.032). **(C)** EBV viral load levels in HIV-negative individuals classified according to EBV genotype (*p* = 0.069).

In this study, all samples (*n* = 155, 100%) showed detection of the EBV genome. The EBV viral load was compared between the HIV-positive and HIV-negative groups. The Kolmogorov (0.158, *p* < 0.0001) and Smirnov tests (0.770, *p* < 0.0001) results showed that the data were not normally distributed. Thus, we used the median EBV viral load to compare HIV-positive and HIV-negative individuals, and non-parametric analytic methods were employed. The median log10 of EBV viral load was 4.23 copies/ml [interquartile range (IQR): 3.76–4.46] in PWH and 3.84 copies/ml (IQR: 3.74–4.02) in the HIV-negative group. The EBV viral load in PWH was higher than that in HIV-negative individuals, and it was statistically significant (*p* = 0.004; (Figure 3A)). In HIV-positive individuals, the median log10 of EBV viral load was 4.25 copies/ml (IQR: 3.83–4.47) in EBV genotype 1, 3.94 copies/ml (IQR: 3.67–4.38) in EBV genotype 2, and 3.70 copies/ml (IQR: 3.52–4.02) in mixed infection of both genotypes. The EBV viral load in EBV genotype 1 was higher than that in EBV genotype 2, and there was a mixed infection of both genotypes. It was found that there was a statistically significant difference in EBV viral load levels among different genotypes of EBV among HIV-positive individuals (*p* = 0.032; (Figure 3B)). In HIV-negative individuals, the median log10 viral load in EBV genotype 1 (median: 3.81 IQR 3.74–4.02) was lower compared with the EBV genotype 2 infected patients (median: 4.10 IQR 3.9–4.20, *p* = 0.069; (Figure 3C)).

## Discussion

To the best of our knowledge, this is the first study that provides baseline information on the molecular characterization of EBV among PWH and HIV-negative individuals in Ethiopia. The main findings of this study are that EBV genotype 1 predominates, followed by EBV genotype 2, and mixed infections of both genotypes. The mixed EBV infections of both genotypes were only found in PWH. In addition, the EBV viral load in PWH was higher than that in HIV-negative individuals. Furthermore, among PWH, the EBV viral load in EBV genotype 1 was higher than EBV genotype 2 and mixed infection of both genotypes, whereas in HIV-negative individuals, the EBV viral load was lower in EBV genotype 1 than EBV genotype 2.

In this study of Ethiopian PWH, EBV genotype 1 was predominant, followed by EBV genotype 2, and there was a mixed infection of both genotypes in PWH only. This finding is comparable with previously reported results in different studies (Correa et al., 2007; Smatti et al., 2018; Monteiro et al., 2020; Tabibzadeh et al., 2020; Liao et al., 2022; Wan et al., 2023). In contrast to our study, a prior report showed that EBV genotype 2 predominated over EBV genotype 1 and mixed infection of both genotypes (Sculley et al., 1990; van Baarle et al., 2000; Santos et al., 2014). None of these studies were performed on samples from PWH individuals in sub-Saharan Africa. Our findings suggest PWH in Ethiopia shows a striking restriction of genotype 1 and co-infection with genotypes 1 and 2.

Immunocompromised patients are more susceptible to acquiring a mixed infection of both EBV genotypes (Bouvard et al., 2009). In our study, only HIV-positive individuals showed evidence of a mixed infection of both genotypes. The presence of mixed infections in PWH has also been previously reported in other studies (Polz et al., 2014; Wan et al., 2023). Characteristics of EBV in immunocompromised and normal control individuals suggest that acquired immunodeficiency leads to more frequent reactivations and higher levels of virus replication (Walling et al., 2003). In people who have lost their prior protective immunity to EBV, mixed infections of both EBV genotypes may accumulate as superinfections. In HIV-negative individuals, there was no detection of mixed EBV infection (Yao et al., 1991; Kunimoto et al., 1992), which is consistent with our findings. However, in contrast to our results, other studies evaluating HIV-negative individuals detected a mixed infection prevalence of between 20 and 53% (Apolloni and Sculley, 1994; Brooks et al., 2000; Srivastava et al., 2000). These studies did not include EBNA3C in the analysis. This variation may be explained by the use of select molecular epidemiology techniques to distinguish EBV genotypes.

The findings of our study revealed that the EBV viral load found in PWH was statistically higher than the EBV viral load in HIV-negative individuals. It was shown that the few free viral particles circulating in plasma might make the EBV viral load a biomarker of an active and replicative infection (Wagner et al., 2002). Furthermore, it has been observed that PWH who have a high EBV viral load have more circulating B cells with the virus. This might increase the likelihood of those EBV-infected cells developing certain lymphomas later on (Fan and Gulley, 2001). Additionally, it appears that immunosuppression is correlated with the level of EBV viral load (Vangipuram and Tyring, 2019).

In our study, there was a difference in EBV viral load levels among different genotypes of EBV among PWH in Ethiopia. A higher viral load of EBV genotype 1 was shown in HIV-positive individuals, while the viral load of EBV genotype 2 was higher in the HIV-negative group. This finding is comparable to a previous study (Pereira et al., 2022). In contrast to our study, a study of PWH in China found higher EBV viral loads to be associated with EBV genotype 2 (Wan et al., 2023). The geographic context may play an important role in the distribution of genotype frequency in PWH.

One limitation of our study is that it was limited to study participants 18 years of age and older, where most of the participants were seropositive, and a similar study with a younger group is needed. Secondly, since the number of samples from HIV-negative participants is typically smaller than those from HIV-positive participants, further study is required to determine better assays with larger sample sizes.

## Conclusion

The study findings revealed that EBV genotype 1 was the predominant genotype, followed by EBV genotype 2 in Ethiopian patients with HIV infection. A mixed infection was seen exclusively in PWH. The viral load of EBV was greater in PWH in general, and specifically, a higher viral load was detected for EBV genotype 1 in PWH, whereas it was higher for EBV genotype 2 in HIV-negative patients. Understanding the genotype characterization of EBV in PWH is essential for developing new and innovative strategies for preventing and treating EBV-related complications in this population.

## Data availability statement

The authors acknowledge that the data presented in this study must be deposited and made publicly available in an acceptable repository, prior to publication. Frontiers cannot accept a manuscript that does not adhere to our open data policies.

## Ethics statement

The studies involving humans were approved by Addis Ababa University, Aklilu Lemma Institute of Pathobiology-Institutional Review Board (protocol number: ALIPB - IRB # 60/2013/21). The studies were conducted in accordance with the local legislation and institutional requirements. The participants provided their written informed consent to participate in this study.

## Author contributions

KZ: Conceptualization, Data curation, Formal analysis, Investigation, Methodology, Writing – original draft, Writing – review & editing. ST: Data curation, Formal analysis, Investigation, Methodology, Writing – review & editing. AH: Formal analysis, Methodology, Writing – review & editing. EA: Formal analysis, Investigation, Methodology, Writing – review & editing. CW: Formal analysis, Investigation, Methodology, Writing – review & editing. AA: Formal analysis, Writing – review & editing. WA: Formal analysis, Methodology, Writing – review & editing. GY: Formal analysis, Methodology, Writing – review & editing. RB: Conceptualization, Funding acquisition, Methodology, Project administration, Resources, Writing – review & editing. TA: Conceptualization, Formal analysis, Methodology, Supervision, Writing – review & editing. NB: Conceptualization, Formal analysis, Methodology, Writing – review & editing.

## Figures and Tables

**Table:** 

DNA	Deoxyribonucleic acid
DMSO	Dimethyl sulfoxide
*EBNA1*	Epstein–Barr nuclear antigen 1
*EBNA2*	Epstein–Barr nuclear antigen 2
*EBNA3C*	Epstein–Barr nuclear antigen 3C
EBV	Epstein–Barr virus
EBV genotype 1	Epstein–Barr virus genotype 1
Mixed infection	Epstein–Barr virus genotype 1 and 2
EBV genotype 2	Epstein–Barr virus genotype 2
GC	Gastric carcinoma
HL	Hodgkin lymphoma
HHV-8	Human herpesvirus-8
HIV	Human immunodeficiency viruses
LMP	Latent membrane proteins
NPC	Nasopharyngeal carcinoma
NHL	Non-Hodgkin lymphoma
PWH	People with HIV
PBMCs	Peripheral blood mononuclear cells
PBS	Phosphate buffer saline
PCR	Polymerase Chain Reaction
qPCR	Quantitative Polymerase Chain Reaction
RNA	Ribonucleic acid
TASH	Tikur Anbessa Specialized Hospital
